# Frequent tracheal suctioning is associated with extubation failure in patients with successful spontaneous breathing trial: a single-center retrospective cohort study

**DOI:** 10.1186/s40981-022-00495-7

**Published:** 2022-01-13

**Authors:** Junpei Haruna, Hiroomi Tatsumi, Satoshi Kazuma, Aki Sasaki, Yoshiki Masuda

**Affiliations:** 1 Department of Intensive Care Medicine, School of Medicine, Sapporo Medical University, South-1, West-16, Chuo-ku, Sapporo, Hokkaido 060-8543 Japan; 2 Department of Nursing, Sapporo Medical University Hospital, Sapporo, Japan

**Keywords:** Reintubation, tracheal suctioning, intensive care unit

## Abstract

**Background:**

Extubation failure, i.e., reintubation in ventilated patients, is a well-known risk factor for mortality and prolonged stay in the intensive care unit (ICU). Although sputum volume is a risk factor, the frequency of tracheal suctioning has not been validated as a predictor of reintubation. We conducted this study to examine whether frequent tracheal suctioning is a risk factor for reintubation.

**Patients and methods:**

We included adult patients who were intubated for > 72 h in the ICU and extubated after completion of spontaneous breathing trial (SBT). We compared the characteristics and weaning-related variables, including the frequency of tracheal suctioning between patients who required reintubation within 24 h after extubation and those who did not, and examined the factors responsible for reintubation.

**Results:**

Of the 400 patients enrolled, reintubation was required in 51 (12.8%). The most common cause of reintubation was difficulty in sputum excretion (66.7%). There were significant differences in sex, proportion of patients with chronic kidney disease, pneumonia, ICU admission type, the length of mechanical ventilation, and ICU stay between patients requiring reintubation and those who did not. Multivariate analysis showed frequent tracheal suction (> once every 2 h) and the length of mechanical ventilation were independent factors for predicting reintubation.

**Conclusion:**

We should examine the frequency of tracheal suctioning > once every 2 h in addition to the length of mechanical ventilation before deciding to extubate after completion of SBT in patients intubated for > 72 h in the ICU.

## Background

Extubation failure, i.e., reintubation, is a known risk factor for mortality and prolonged intensive care unit (ICU) stay in patients who receive mechanical ventilation in the ICU [1, 2]. Incidence of reintubation has been reported to be approximately 10%, and therefore, we should keep in mind the risk of reintubation and aim for safe extubation [3, 4]. Epstein has demonstrated that patients with advanced age, anemia, hypoalbuminemia, and chronic respiratory failure, and those undergoing highly invasive surgery carry a risk for reintubation [5]. Moreover, clinical tools for evaluating successful extubation, such as the rapid shallow breathing index (RSBI) [6], are available for spontaneous breathing trials (SBTs). Therefore, extubation should be tried carefully after confirming favorable results of SBT in mechanically ventilated patients. However, reintubations sometimes occur despite following established protocols [7].

The main causes of reintubation are difficulty in sputum excretion and airway obstructions [5]. Sputum excretion is challenging owing to the amount of sputum available for extraction and the ability to discharge it [8, 9]. Although it is difficult for ICU healthcare workers to quantitatively evaluate the amount of airway secretion when performing trachea suctioning, the frequency of tracheal suction during mechanical ventilation may be associated with the amount of tracheal secretions [10]. We hypothesized that the frequency of tracheal suctioning is associated with the amount of tracheal secretions. This study, therefore, elucidates whether reintubation is associated with the frequency of tracheal suctioning before extubation.

## Methods

We performed a single-center, retrospective, observational study in a university hospital. We collected data from patients admitted to the ICU between January 2011 and December 2017. The study design and protocol were approved by the Institutional Review Board (IRB) of Sapporo Medical University (IRB authorized number: 322-266). Owing to the observational nature of this study, the information was released on an opt-out basis.

We included patients (≥ 18 years) who had been intubated for > 72 h in the ICU of Sapporo Medical University Hospital and extubated after confirming successful SBT results defined as follows: respiratory rate < 30/min, SpO_2_ > 94%, heart rate < 140 bpm, no arrhythmia, no excessive increase in blood pressure and no effort breathing, under continuous positive airway pressure ≤ 5 cmH_2_O or pressure support ≤ 5 cmH_2_O or T-piece for ≥ 30 min with inspiratory oxygen concentration (FIO_2_) ≤ 0.4. SBT was performed when the patient fulfilled the condition described in Table 1, and the ultimate decision to extubate was made by the intensive-care clinician based on SBT data, as well as the patient’s hemodynamic stability, responsiveness, ability to follow commands, the strength of cough, and the ability to clear secretions. The exclusion criteria were patients after cardiovascular surgery or those with a tracheostomy. Extubation failure was defined as reintubation within 24 h after extubation.

**Table 1:** Criteria for initiating SBT

(1) Oxygenation	
SpO_2_ >90% under FIO_2_≤0.5 and PEEP≤8 cmH_2_O	
(2) Circulation	
No acute myocardial ischemia or serious arrhythmia	
Heart rate <140 bpm	
DOA ≦ 5μg/kg/min, DOB ≦ 5μg/kg/min, NAD ≦ 0.05μg/kg/min	
(3) Ventilation	
Tidal volume>5 ml/IBW	
Respiratory minute volume<15 L/min	
Rapid shallow breathing index < 105/min/L	
No respiratory acidosis (pH<7.25)	
Normal breathing pattern	
No respiratory effort	
(4) General condition	
No serious electrolyte disorders	
No severe anemia	
No serious fluid overload	

*SBT* spontaneous breathing trial, *DOA* dopamine, *DOB* dobutamine, *NAD* noradrenaline, *IBW* ideal body weight

Information obtained from electronic medical records included age, sex, underlying disease, Charlson Comorbidity Index (CCI), patient category at ICU admission (postoperative or medical), ICU length of stay (LOS), Acute Physiology and Chronic Health Evaluation (APACHE) II score at ICU admission, Sequential Organ Failure Assessment score at ICU admission, ventilation days, 28-day mortality, delirium, RSBI, and frequency of tracheal suctioning. The primary outcome was the association between reintubation within 24 h after extubation and tracheal suctioning frequency (more than once every 2 h for up to 12 h) [5, 11] before extubation.

The definition of tracheal suctioning is as follows.

1. The patient’s effortful breathing is increased (increased respiratory workload findings).
2. Secretions are visible in the tracheal tube.
3. Coarse crackles that suggest the presence of secretions from the trachea to the right and left main bronchi are heard on chest auscultation, or there is a decrease in breath sounds on chest auscultation.
4. Palpation of the chest wall reveals vibrations associated with the movement of gas.
5. Aspiration.
6. Blood gases and blood oxygen saturation rate (SpO_2_) show hypoxemia.
7. An increase in airway pressure or a decrease in ventilation volumes.

Reintubation was performed when the patient had one or more of the following criteria: clinical signs of increased respiratory effort, upper airway obstruction, respiratory acidosis, hypoxemia (SpO_2_<90%), decreased consciousness with unprotected upper airway (GCS<8), severe tachycardia, and continued tachypnea.

### Statistical analysis

Categorical variables were expressed as numbers and percentages. Continuous variables were expressed as means and standard deviations. Chi-square tests were used for the nominal variables. The Mann-Whitney *U* test was used for continuous variables. We hypothesized that the frequency of tracheal suctioning was an independent risk factor for reintubation. To evaluate this hypothesis, logistic regression analysis was performed to examine the odds ratio of the frequency of tracheal suctioning, adjusting for confounding factors that contribute to reintubation. We selected covariates based on the previous literature and clinical experience. Sex, CCI [7, 12], underlying pneumonia, APACHE II score at ICU admission [13], and ventilation days [12] were selected as confounding factors. The results of the multivariable analysis are shown with odds ratios (ORs), 95% confidence intervals (CIs), and *p*-values. Statistical significance was set at *p* <0.05. Statistical analyses were performed using SPSS Statistics version 27 (IBM Corp., Armonk, NY, USA).

## Results

**Fig. 1:**
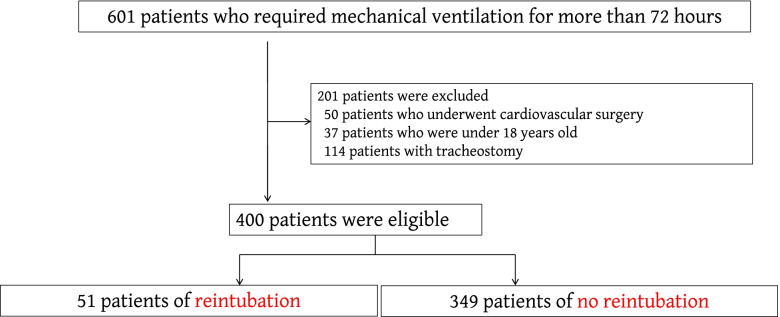
Flow diagram of this study

During the study period, 601 patients received ventilator support for more than 72 h. Fifty-one patients required reintubation within 24 h after extubation (reintubation group) (Fig. 1). The demographics of the patients and a comparison of vital signs and respiratory parameters in the reintubation and no reintubation groups are shown in Table 2.

**Table 2:** Demographics of patients with and without reintubation

	Reintubation group	No reintubation group	*P value*
	*n*=51	*n*=349	
Age (years), mean±SD	65.2±13.3	64.3±14.2	0.878
Gender, male, *n* (%)	24(47.1)	235(67.3)	0.007
Comorbidities			
Immune suppression	4(7.8)	26(2.9)	1
Chronic liver disease	6(11.8)	27(7.7)	0.4
Chronic kidney disease	6(11.8)	2(5.7)	<.001
Chronic heart failure	5(9.8)	60(17.2)	0.225
Charlson Comorbidity Index	1.6±1.8	1.7±1.9	0.73
Underlying diseases			
Pneumonia	17(33.3)	47(13.5)	<.001
Sepsis	7(13.7)	36(10.3)	0.468
ARDS	4(7.8)	16(4.6)	0.303
heart failure	3(5.9)	12(3.4)	0.421
Miscellaneous	20(39.2)	237(67.9)	<.001
Admission type			
Surgical	18(35.3)	179(51.0)	0.036
Medical	33(64.7)	170(49.0)	0.036
SOFA at ICU admission, mean±SD	6.4±2.5	7.0±2.8	0.048
APACHE II at ICU admission, mean±SD	21.1±6.2	20.8±5.8	0.675
Delirium, *n* (%)	14 (27.5)	105 (30.1)	0.746
RSBI	39.6±12.9	38.6±16.5	0.322
Ventilation days (day)	13.3±11.8	5.0±2.7	<.001
Time to reintubation, mean±SD	492±470.4	-	
ICU LOS (days), mean±SD	14.2±12.5	8.7±8.2	<.001
ICU mortality, *n* (%)	2(3.9)	10(2.9)	0.223
28-day mortality, *n* (%)	6(11.8)	17(4.9)	0.097
Reason of reintubation, *n* (%)			
Difficulty in sputum excretion	30 (58.8)	-	
Hypoxemia	9 (17.6)	-	
Airway obstruction	6 (11.8)	-	
Tachypnea	4 (7.8)	-	
Consciousness disturbance	1(2.0)	-	
Hemosputum	1(2.0)	-	

*ICU* intensive care unit, *APACHE II* Acute Physiology and Chronic Health Evaluation, *SOFA* Sequential Organ Failure Assessment, *ARDS* acute respiratory distress syndrome, *LOS* length of stay, *RSBI* rapid shallow breathing index

Sex (male), admission type, ventilation days, and ICU LOS were significantly higher in the reintubation group. Furthermore, comorbidity of chronic kidney disease and underlying pneumonia was significantly higher in the reintubation group. The mean time to reintubation was 492 min. The most common cause of reintubation was difficulty in sputum excretion, followed by hypoxemia and airway obstruction.

The results of the logistic regression analysis adjusted for the pre-defined covariates to examine the effect of the frequency of tracheal suctioning on reintubation are shown in Table 3.

**Table 3:** Odds ratios regarding variables for extubation failure

	Regression coefficient	OR	95%CI	*P value*
Male	0.827	2.287	1.03–5.08	0.042
CCI	−0.133	0.875	0.70–1.09	0.242
Pneumonia	−0.585	0.557	0.225–1.381	0.207
APACHE II	−0.004	0.996	0.93–1.06	0.899
Tracheal suction more than once every 2 h	2.365	10.65	4.60–24.62	<.001
Ventilation days	0.272	1.31	1.19–1.44	<.001

*OR* odds ratio, *CCI* Charlson Comorbidity Index, *APACHE II* Acute Physiology and Chronic Health Evaluation

A higher frequency of tracheal suctioning, defined once every 2 h up to 12 h before the extubation trial, was an independent factor associated with reintubation (OR = 10.65, 95% CI = 4.60–24.62, *p*<.001). Moreover, ventilation days were also an independent factor associated with reintubation (OR, 1.33; 95% CI = 1.19–1.45, *p*<.001).

## Discussion

In this study, we evaluated the association between reintubation and the frequency of tracheal suctioning in ventilated patients for more than 72 h and fulfilled extubation criteria such as SBT. The reintubation rate after extubation with successful SBT was 12.8%, which was similar to a previous report [14]. The most common cause of reintubation was hypoxemia, followed by difficulty in sputum excretion. We found that tracheal suction more than once every 2 h was an independent factor associated with reintubation. The results of this study suggest that it may be useful to include the frequency of tracheal suctioning before extubation as one of the criteria for extubation in ventilator management.

In general, airway problems are a risk factor for reintubation [15]. One of the most common airway problems is the inability to clear airway secretions [14, 16]. The clearance of airway secretions has two components: the intensity of cough and the volume of airway secretions. An association between cough intensity and reintubation has been reported in relation to a decrease in peak expiratory flow (PEF); however, the procedure for measuring PEF [15] is cumbersome and difficult to perform during daily clinical practice. Conversely, it has been reported that a high volume of airway secretion is associated with reintubation [2, 14, 17, 18]. However, it is difficult for ICU healthcare workers to measure the amount of airway secretions when performing tracheal suctioning, thereby limiting the prediction of reintubation based on the amount of sputum secreted. Another way for ICU healthcare workers to determine the amount of airway secretions is the frequency of tracheal suctioning. It has been reported that intensivists use lower levels of airway secretion as an adjunctive indicator of successful extubation [14, 19]. In this study, the frequency of tracheal suctioning was associated with reintubation. Therefore, it may be useful to decide extubation based on the frequency of tracheal suctioning. Respiratory physiotherapy may be useful to prevent reintubation in patients requiring frequent tracheal suctioning to prevent respiratory complications and for excretion training of airway secretions [4, 20].

Our results showed that the duration of mechanical ventilation was longer in the reintubation group than in the no reintubation group. Prolonged mechanical ventilation has been reported to negatively affect respiratory and limb (or extremities) muscle strength [21, 22] and is thought to result in ICU-acquired weakness. This study did not evaluate the onset of ICU-acquired weakness; however, it is important to provide multidisciplinary interventions such as early rehabilitation from the start of mechanical ventilation to achieve no reintubation. Particularly, when extubating patients with prolonged mechanical ventilation and a high volume of sputum, it should be considered that even with a successful SBT, extubation with respiratory physiotherapy is a strategy to reduce reintubation.

This study had several limitations. First, this is a retrospective, single-center, observational study. Therefore, our results may not be generalizable and need to be validated externally in future studies using a different dataset. Second, we used the nursing records obtained from an electronic patient information system, which may not reliably reflect actual clinical symptoms.

## Conclusion

We found that a higher frequency of tracheal suctioning and ventilator days were associated with risk factors for reintubation in critically ill patients on mechanical ventilation. It is necessary to introduce protocols and other measures, such as clearer extubation criteria, to reduce reintubation in this population.

## Data Availability

The datasets analyzed during the current study are available from the corresponding author upon reasonable request.

## Abbreviations

ARDS: Acute respiratory distress syndrome
AW: Acquired weakness
APACHE: Acute Physiology and Chronic Health Evaluation
CCI: Charlson Comorbidity Index
CI: Confidence interval
FIO_2_: Fractional inspired oxygen concentration
ICU: Intensive care unit
LOS: Length of stay
OR: Odds ratio
PEF: Peak expiratory flow
RSBI: Rapid shallow breathing index
SBT: Spontaneous breathing trial
SOFA: Sequential Organ Failure Assessment
SpO_2_: Blood oxygen saturation rate
